# Association of the Hepatocyte Growth Factor Gene with Keratoconus in an Australian Population

**DOI:** 10.1371/journal.pone.0084067

**Published:** 2014-01-08

**Authors:** Srujana Sahebjada, Maria Schache, Andrea J. Richardson, Grant Snibson, Mark Daniell, Paul N. Baird

**Affiliations:** 1 Centre for Eye Research Australia, Melbourne, Australia; 2 The University of Melbourne, Melbourne, Australia; 3 Royal Victorian Eye and Ear Hospital, Melbourne, Australia; Osaka University Graduate School of Medicine, Japan

## Abstract

**Purpose:**

A previous study has indicated suggestive association of the hepatocyte growth factor (*HGF*) gene with Keratoconus. We wished to assess this association in an independent Caucasian cohort as well as assess its association with corneal curvature.

**Participants:**

Keratoconus patients were recruited from private and public clinics in Melbourne, Australia. Non-keratoconic individuals were identified from the Genes in Myopia (GEM) study from Australia. A total of 830 individuals were used for the analysis including 157 keratoconic and 673 non keratoconic subjects.

**Methods:**

Tag single nucleotide polymorphisms (tSNPs) were chosen to encompass the hepatocyte growth factor gene as well as 2 kb upstream of the start codon through to 2 kb downstream of the stop codon. Logistic and linear regression including age and gender as covariates were applied in statistical analysis with subsequent Bonferroni correction.

**Results:**

Ten tSNPs were genotyped. Following statistical analysis and multiple testing correction, a statistically significant association was found for the tSNP rs2286194 {p = 1.1×10-^3^ Odds Ratio 0.52, 95% CI - 0.35, 0.77} for keratoconus. No association was found between the 10 tSNPs and corneal curvature.

**Conclusions:**

These findings provide additional evidence of significant association of the *HGF* gene with Keratoconus. This association does not appear to act through the corneal curvature route.

## Introduction

Keratoconus (KC) is a common corneal condition characterised by a progressive corneal thinning resulting in corneal protrusion, irregular astigmatism and decreased vision [1]. It accounts for some 31% of corneal grafts in Australia [2]. There is no medical treatment to halt the progression of KC apart from stiffening of the cornea with riboflavin/UVA-cross-linking. Other rare forms of surgical treatment include epikeratoplasty, photorefractive keratectomy, or intra corneal rings [3], [4], [5]. All these techniques correct the refractive error of KC but do not treat the underlying cause of the corneal weakness and thinning, and therefore cannot stop the progression of KC. However, recent clinical trials using corneal cross linking, currently provide one of the best strategies to retard the progression of KC [6].

KC is a heterogeneous disorder believed to be caused by both genetic and environmental factors [7]. KC has been reported to occur through several routes which include eye rubbing [8]; allergy [9], and contact lens wear [10] as well as in those with a family history [11]. A number of studies have indicated a genetic involvement in KC including twin studies [12], familial aggregation studies [13] and formal genetic analyses [14] and genetic linkage has been reported to multiple loci [15].Despite the extensive genetic research in KC over the past decades, only a few genes have been reported including *VSX1*, the visual system homeobox-1 gene [16], [17], [18], [19], [20]; superoxide dismutase 1 (*SOD1*) gene [21]; interleukin 1B (*IL1B*) [22] gene; two collagen genes, *COL4A3* and *COL4A4* [23] and lysyl oxidase (*LOX*) [24] - although not all studies support these findings [25], [26], [27], [28], [29]. Other gene loci have been implicated through the use of a recent Genome Wide Association Study (GWAS) on central corneal thickness where Lu *et al.* [30] identified 6 genetic variants associated with KC. This study implicated the *FNDC3B* (rs4894535), *MPDZ-NF1B* (rs132183), *RXRA-COL5A1* (rs1536482), *COL5A1* (rs7044529), *FOXO1* (rs2721051) and *BANP-ZNF469* (rs9938149) genes. Additionally, an association study using a combined total of 933 KC cases and 4164 controls from Australia, the USA and Northern Ireland has also identified a putative association with single nucleotide polymorphisms (SNPs) in the Hepatocyte growth factor (*HGF*) gene [15].This involved both GWAS followed by replication in additional cohorts. Association was identified with SNP rs3735520 (p = 9.9×10-^7^) and rs17501108 (p = 9.9×10-^5^) with KC. Although the SNPs in this study did not reach genome wide significance, it did indicate the *HGF* gene as a strong candidate gene for further analysis.

Given the paucity of replication studies confirming genetic associations with KC, we wished to undertake a replication study of the *HGF* gene. We undertook a case-control study to investigate the association of this gene with KC as well as corneal curvature in a cohort consisting of Caucasian individuals of European descent.

## Methods

### Participants

All patients for this study were recruited from public clinics at the Royal Victorian Eye and Ear Hospital (RVEEH), private rooms, optometry clinics or consenting general public with KC. A patient information sheet, consent form, privacy statement and patient rights were provided to all individuals participating in the study. Non-keratoconic subjects were obtained through the Genes in myopia (GEM) study where a similar testing protocol was used with the administration of a general questionnaire, comprehensive eye examination and blood collection. The methodology for the GEM study has been published elsewhere [31].

### Protocol

KC patients were required to complete a study questionnaire, clinical examination and complete details of family history of disease. A blood/saliva sample was also collected. The entire procedure lasted less than an hour. The Study protocol was approved by the Royal Victorian Eye and Ear Hospital Human Research and Ethics Committee (Project # 10/954H). Written informed consent was obtained from each participant after explanation of the nature and possible consequences of the study. This protocol followed the tenets of the Declaration of Helsinki and all privacy requirements were met.

### Inclusion and exclusion criteria

Individuals with KC of European background, presenting to clinics/private practices were invited to participate in the study. KC was diagnosed on the basis of the presence of one or more of the following:

1. an irregular cornea, as determined by distortion of keratometric mires/and or orbscan/pentacam images
2. Scissoring of the retinoscopic reflex;
3. demonstrating at least one bio microscopic sign, including Vogt's striae, Fleischer's ring or corneal thinning and scarring typical of KC.
  And one or more of the following changes in topographic map-
4. Focal steepening of areas greater than 46–47 dioptres (D), located in the cone protrusion zone surrounded by concentric decreasing power zones.
5. Angling of the hemi meridians, exceeding 20 or 30 degrees, in the bow tie pattern
6. Inferior-superior asymmetry greater than 1.4D within the mid peripheral cornea

Potential subjects with non-KC ocular disease in both eyes such as keratectasia, corneal degenerations, macular disease, and optic nerve disease (e.g., optic neuritis, optic atrophy) were excluded from the study. Individuals of non-European descent were not included in the study.

### tSNP selection and genotyping

A total of 11 tag SNPs (tSNPs) were selected that encompassed a region beginning 2 kb upstream of the start codon through to 2 kb downstream of the stop codon of the Hepatocyte growth factor gene. tSNPs were selected using the pairwise algorithm and a CEU population. A strong LD tagging criteria of r^2^>0.8 was used and a minor allele frequency (MAF) of 0.01 was chosen. tSNPs were sourced using hap MAP (http://www.hapmap.org) (hapMAP genome browser release #24 Phase 1+2 full dataset NCBI Build 36 assembly dbSNPb126). The tSNPs chosen for this study included rs5745752, rs2286194, rs5745696, rs5745692, rs5745687, rs17155414, rs12707453, rs1019012, rs5745660, rs5745627 and rs5745616 (Figure S1). Primer information relating to amplification of these tSNPs is presented in Table S1. Genotyping was performed on the Mass Array platform (SEQUENOM, San Diego, CA) at the Murdoch Children's Research Institute, Melbourne.

## Statistical Analysis

All statistical analysis were performed using PLINK v1.04 (http://pngu.mgh.harvard.edu/~purcell/plink/). KC associations were analysed using a logistic regression model and corneal curvature was analysed using a linear regression model. For all analysis, age and sex were used as covariates. Multiple testing was accounted for using Bonferroni correction. All tSNPs were assessed for deviation from Hardy-Weinberg equilibrium with a p-value of 0.05 being the cut off. Only tSNPs passing the Hardy-Weinberg equilibrium (HWE) test were used for statistical analysis. Power calculations were performed using Quanto v1.2.4 using an allele frequency of 0.15 (averaged from the genotyped tSNPs) and an alpha of 0.05.

### *In silico* analysis

Detection of evolutionary conservation across species, enhancer elements, miRNA sequences and transcription factor binding sites was undertaken using 0.5 kb of genomic sequence upstream and downstream of rs2286194. Evolutionary conservation analysis was undertaken using the ECR browser (http://ecrbrowser.dcode.org) [32], enhancer element detection using the VISTA Enhancer Browser (http://enhancer.lbl.gov) [33], miRNA detection using miRBase (http://enhancer.lbl.gov) [33] and transcription factor binding site prediction analysis using Match v1.0 (www.gene-regulation.com/index2.html).

## Results

### Clinical Data on Participants

A total of 830 individuals [354 (43%) males, 476 females mean age = 49.85±16.30 years] including 157 individuals with KC [93 (59%) males, 64 females; mean age = 37.81±15.65 years] and 673 non KC subjects (260 males, 413 females; mean age = 52.67±15.15 years) were recruited into the study. There was a very high correlation between measures for right and left eyes (r^2^>0.99) therefore only data from the right eye were used in the analysis. The KC eyes were significantly steeper and more myopic (p<0.05) compared to the non KC eyes. There was no significant difference between axial length (p = 0.77) and anterior chamber depth (0.97) between KC and non KC individuals. Clinical characteristics for each group are shown in Table 1.

**Table 1:** Clinical characteristics of the study cohort used in the assessment of the Hepatocyte Growth Factor (*HGF*) Gene.

	Keratoconus Mean ±SD	Non Keratoconus Mean ± SD
**Spherical Equivalent (D** * **)**	−4.54±4.71	−1.96±3.77
**Axial Length (mm)**	24.32±1.55	24.64±1.71
**Corneal Curvature (D** * **)**	49.28±8.19	42.69±2.54
**Anterior Chamber Depth (mm)**	3.52±0.57	3.49±0.41

Dioptres.

A total of 11 tSNPs were genotyped including rs5745752, rs2286194, rs5745696, rs5745692, rs5745687, rs17155414, rs12707453, rs1019012, rs5745660, rs5745627 and rs5745616. The genotyping call rate for all SNPs was >96% after we excluded rs5745660 which had a call rate of 85% (Table S2). Genetic association was performed for the 10 tSNPs between KC subjects and non KC subjects. A Bonferroni corrected *P* value of 0.05/10 = 0.005 was applied as appropriate for independent tSNPs because of the multiple comparisons made in this study.

Only the “A” minor allele of tSNP rs2286194 (p = 1×10^−3^) (odds ratio (OR) 0.52, 95% confidence intervals (95% CI) 0.35, 0.77) was significantly associated with inverse risk of KC compared to non KC (Table 2). In addition, rs5745752 appeared nominally significant (OR 1.58, 95% CI 1.19–2.09) (p = 8.1×10^−3^) with associated increased risk of KC (Table 2). Quantitative analysis of the 10 tSNPs for corneal curvature using linear regression methodologies did not show significant association with KC although the tSNP rs2286194 appeared as the most significant (p = 0.049) (Table 3).

**Table 2:** Logistic regression analysis for the assessment of the Hepatocyte Growth Factor (*HGF*) Gene with corneal curvature and keratoconus.

tSNP*	Position	Minor Allele			Odds Ratio	P**
		Name	Freq. (controls)	Freq. (cases)	(95% CI)	
**rs2286194**	81,193,385	A	0.209	0.131	0.52 (0.35–0.77)	0.0011
**rs5745752**	81,173,396	A	0.291	0.409	1.58 (1.19 –2.09)	0.0081
**rs5745696**	81,195,996	A	0.068	0.058	0.70 (0.39–1.23)	0.2134
**rs5745692**	81,196,202	C	0.028	0.029	1.29 (0.58–2.89)	0.5279
**rs5745687**	81,196,987	A	0.069	0.073	1.06 (0.64–1.78)	0.8118
**rs17155414**	81,200,690	A	0.219	0.229	1.09 (0.79–1.53)	0.5837
**rs12707453**	81,207,355	G	0.199	0.218	1.05 (0.75–1.47)	0.7755
**rs1019012**	81,208,710	G	0.2	0.222	1.17 (0.84–1.64)	0.3481
**rs5745627**	81,230,474	C	0.031	0.026	0.91 (0.39–2.14)	0.8296
**rs5745616**	81,236,292	A	0.235	0.257	1.09 (0.80–1.49)	0.5664

tSNPs: Tag Single Nucleotide Polymorphism.

P value for statistical significance is 0.05/10 = 0.005.

**Table 3:** Linear regression analysis for single nucleotide polymorphisms in the *HGF* gene and corneal curvature.

tSNP*	Position	Minor Allele		Beta	S.E.M**	P***
		Name	Freq.			
**rs2286194**	81,193,385	A	0.209	−0.739	0.375	0.0492
**rs5745752**	81,173,396	A	0.291	0.044	0.296	0.8829
**rs5745696**	81,195,996	A	0.291	−1.576	0.579	0.0067
**rs5745692**	81,196,202	C	0.068	0.044	0.296	0.8829
**rs5745687**	81,196,987	A	0.028	−0.897	0.567	0.1142
**rs17155414**	81,200,690	A	0.069	0.045	0.363	0.901
**rs12707453**	81,207,355	G	0.219	−0.346	0.366	0.3445
**rs1019012**	81,208,710	G	0.199	0.526	0.359	0.1429
**rs5745627**	81,230,474	C	0.039	−1.317	0.879	0.1348
**rs5745616**	81,236,292	A	0.031	−0.403	0.342	0.239

tSNP: Tag single nucleotide polymorphism.

SEM: standard error of the mean.

P value for statistical significance is 0.05/10 = 0.005.

### *In Silico* Analysis

An analysis of evolutionary conservation around rs2286194 indicated very high conservation between the human DNA sequence and that of the Rhesus monkey (92.2%). Moderate conservation was observed between the human DNA sequence and that of the rat (84.1%), chicken (81.4%), dog (77.8%), cow (77.4%), mouse (76.8%), opossum (74.4%) and frog (73.6%) A search for putative transcription factor binding sites and enhancer elements in the region did not detect any. A search for miRNA sequences in the region detected 32 known miRNA elements.

## Discussion

**Figure 1:**
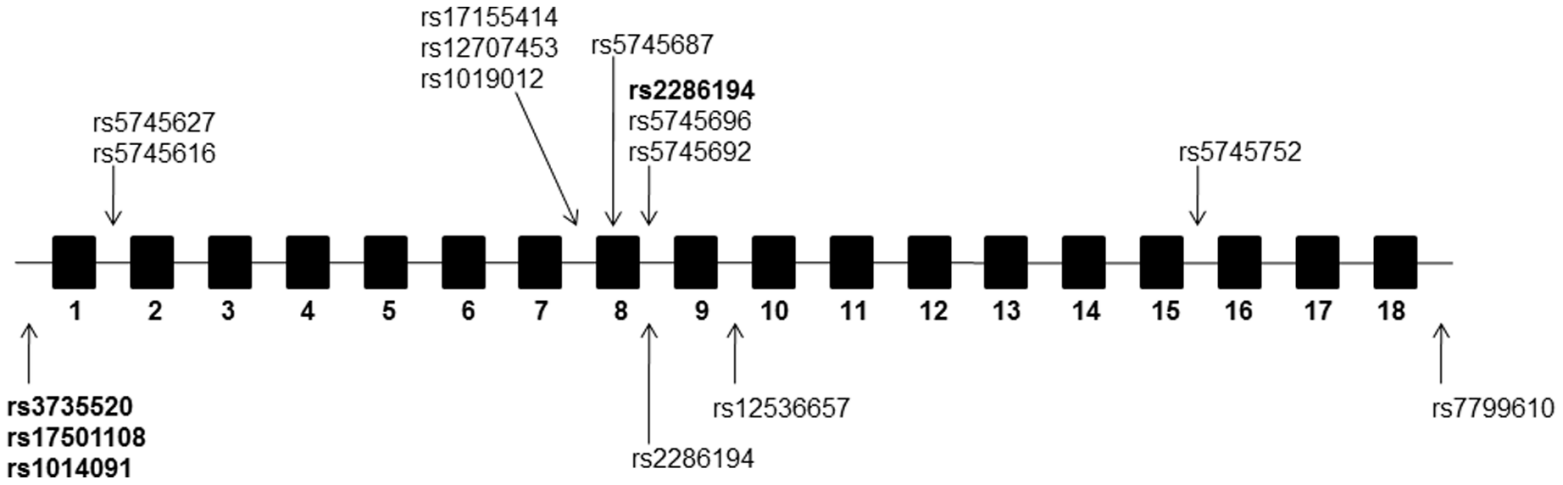
Schematic representation of tSNP locations used in the assessment of the *HGF* gene. Boxes represent exons and horizontal lines represent introns, 5′ and 3′ regions of the gene. Arrows above the line indicate tSNPs genotyped in the current study and those below the line represent those described by Burdon *et al.*(84) SNPs with a statistically significant association with keratoconus in each study are indicated in bold. (Note, exon and intron sizes are not to scale).

Our study indicated that the tSNP rs2286194 in the *HGF* gene is inversely and significantly associated with KC although this association does not appear to be derived through a corneal curvature route. A previous GWAS with replication and subsequent meta-analysis indicated that tSNPs in the promoter and upstream region of the *HGF* gene were associated with increased risk of KC [15]. They showed that the tSNPs rs3735520, rs17501108 and rs1014091 of the *HGF* gene were associated with risk of KC with an OR of 1.5 to 2.22 respectively. In contrast, the tSNP rs2286194 reported in the current study was not associated with KC in their study (p = 0.15) [15].Interestingly, the minor A allele of that study reported the risk allele with allele frequencies of 0.167 in controls and 0.204 in cases whereas in the current study it presents as a protective allele with allele frequencies of 0.209 in controls {which is closer to the allele frequency in the Caucasian population of 0.267, as obtained from Hap Map (CAUC1 population)} and 0.131 in cases (Table 2). Figure 1 shows a schematic representation of tSNP location from the current study and from Burdon *et al.* study in the *HGF* gene. While we were not able to directly replicate the previously associated *HGF* tSNPs with KC in the current study, we were able to demonstrate that tSNP (rs228194) in the *HGF* gene appeared as being significantly associated with KC. While an explanation as to why different tSNPs have been identified in the *HGF* gene in the two studies is not clear at this stage, it could point to several genetic variants tagging different protective and risk haplotypes associated with KC or the existence of genetic heterogeneity in the KC population as we used an independent cohort of Europeans. The latter has some precedence as in the Burdon *et al.* study [15], not all cohorts reached statistical significance although they did have OR's in the same direction.

Thus independent studies now report a positive association of tSNPs in the *HGF* gene with KC. The utility of having multiple studies regarding association with a particular gene strengthens the argument that the gene is of importance in keratoconus and unlikely to be a false positive. This is particularly relevant as keratoconus has had many previous false positive associations that could not be confirmed in subsequent studies.

Yanovitch et al, have also reported SNP rs2286194 in the *HGF* gene as having a strong association with mild to moderate myopia and a moderate association with extremely high myopia [34]. rs2286194 occurs between exon 8 and 9 and it is in an intron and to date no functional data are available for this SNP which might provide some insight into its role in disease. As this is a tag SNP, then it may well be the case that another SNP in high LD presents as the causative SNP and sequencing of such regions is most likely to identify such causative variants.

In addition the in-silico analysis in the current study predicted a high evolutionary conservation of the DNA sequence in the region of rs228194 implying that this may play an important role in the function of this gene. This was further substantiated by the presence of multiple miRNA sequences. However additional work is needed to confirm what functional role is played at this role and how it impacts on KC.

The *HGF* gene has been previously reported as being associated with refractive error in multiple populations including myopia in Han Chinese [35] as well as by ourselves for both myopia and hypermetropia in Caucasians [36] and by others in myopia [34]. The refractive power of the eye is determined at least in part by the shape of the cornea, which is severely altered in KC, thus this may suggest an overlap between genetic determinants of these complex ophthalmic conditions. As a consequence we undertook analysis of corneal curvature to assess if the *HGF* gene was associated with this trait. While we could not detect any significant association with the chosen tSNPs (the most significant SNP being rs2286194, p = 0.49), a power calculation suggested that this study had 84% power to detect an OR of 1.6 or greater. The effect size of any such association would be unknown as it has not been undertaken before in KC. Thus while we cannot exclude associations with corneal curvature existing at lower odds ratios, the association of *HGF* with KC does not currently appear at to come via a corneal curvature route.

The strengths of our study design lie in the choice of our cohort and our tSNPs strategy for analysis. We have been careful to include individuals in our cohort that are only of European background, thereby reducing the chance of biased results owing to population admixture. Additionally, none of the KC subjects were obviously related. Utilizing a tSNP approach has also strengthened our study by ensuring that a large proportion of the genetic coverage of the *HGF* gene was obtained. However, the tSNP approach also has limitations in that only common tSNPs (minor allele frequency 10%) were genotyped. The limitation of our study is the relatively small sample size. However, given that this is a relatively rare condition it is similar in size to a number of other single cohort studies as reported by Burdon *et al.* where a positive association was shown for tSNPs rs373550 in two cohorts with 193 and 222 cases as well as the tSNP rs17501108 and the array SNP rs1014091 that were positively associated in 3 cohorts which consisted of 193, 215 and 222 cases [15]. Therefore the sample size would not present as an obvious cause of not being able to replicate the association findings previously reported.

Our study further implicates the *HGF* gene in KC acting via a non-corneal curvature related route. The finding that this gene is expressed in many different tissues of the eye, including the lacrimal glands, tears and cornea [37], and its known role in cell signalling through its receptor *cMET*, all point to a pivotal role for this gene in KC. *HGF* expression is also up-regulated in response to corneal injury in corneal keratinocytes [38].In addition, the inflammatory cytokines IL-6 and TNF have been detected at elevated levels in tears of patients with severe KC [39], [40] and also in eyes with subclinical KC [41].The human and mouse *HGF* gene promoters both contain binding sites for the pro inflammatory cytokine IL-6 [42] and IL-6 promotes *HGF* gene transcription [42], [43].These findings would suggest that IL-6 and *HGF* are important players in KC and further work exploring the functional role of *HGF* as well as its interacting proteins and downstream signalling pathways should be explored in KC.

In conclusion, our study suggests that a common variant in the *HGF* gene is associated with KC, furthering implicating this gene as important for this disease. This is also the first study to investigate possible association of the *HGF* gene with corneal curvature.

## Supporting Information

Figure S1 **Linkage disequilibrium (LD) plot for all the tag single nucleotide polymorphisms of the** ***HGF*** **gene.** This LD plot indicates that there is only one LD block comprising the tSNPs rs17155414, rs12707453, rs1019012. The tSNPs rs2286194 and rs5745616 are not in any LD block.
(DOCX)

Table S1 **Primers used for the amplification of tag single nucleotide polymorphisms of the** ***HGF*** **gene.**
(DOCX)

Table S2 **Raw data of genotyping frequency and tag single nucleotide polymorphisms call rate of the** ***HGF*** **gene.**
(DOCX)
