# Supplementary material for: Association of the Hepatocyte Growth Factor Gene with Keratoconus in an Australian Population
Source: PLoS One. 2014 Jan 8;9(1):e84067. doi: 10.1371/journal.pone.0084067 (PMC3885514; doi:10.1371/journal.pone.0084067)
Supplement: Figure S1 — Linkage disequilibrium (LD) plot for all the tag single nucleotide polymorphisms of the HGF gene. This LD plot indicates that there is only one LD block comprising the tSNPs rs17155414, rs12707453, rs1019012. The tSNPs rs2286194 and rs5745616 are not in any LD block. (DOCX) [file pone.0084067.s001.docx]

**Supplementary Figure S1.Linkage disequilibrium (LD) plot for all the tag single nucleotide polymorphisms of the *HGF* gene**

**
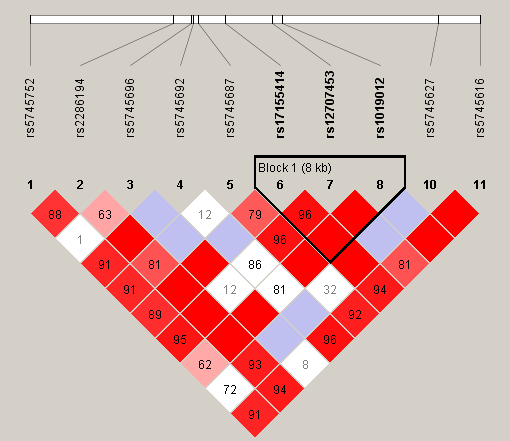
**

**This LD plot indicates that there is only one LD block comprising the tSNPs rs17155414, rs12707453, rs1019012. The tSNPs rs2286194 and rs5745616 are not in any LD block.**
