# Supplementary material for: Association of the Hepatocyte Growth Factor Gene with Keratoconus in an Australian Population
Source: PLoS One. 2014 Jan 8;9(1):e84067. doi: 10.1371/journal.pone.0084067 (PMC3885514; doi:10.1371/journal.pone.0084067)
Supplement: Table S1 — Primers used for the amplification of tag single nucleotide polymorphisms of the HGF gene. (DOCX) [file pone.0084067.s002.docx]

**Supplementary Table S1. Primers used for the amplification of tag single nucleotide polymorphisms of the *HGF* gene**

| **tSNP ID** | **ForwardPCR primer** | **^Reverse^ PCR primer** | **Extension primer** |
| --- | --- | --- | --- |
| **rs5745616** | ACGTTGGATGTGTCTCCATCTGGGCATTTG | ACGTTGGATGGGGAGAAGGCAGTGAATATC | ATATCAAGGGTAGGC |
| **rs1019012** | ACGTTGGATGGTTCCAAACCAATCTTTAGG | ACGTTGGATGAGCTAGCCAGTGTCATAGTC | ATTTCTTGCCTCTCTCA |
| **rs5745752** | ACGTTGGATGCATATGTTACATGCTGTAT | ACGTTGGATGGCAAATACCTTTCTCTCTAT | AGAGAGACCCAAACGCA |
| **rs5745692** | ACGTTGGATGTTCCTCATAACAAGGTGGAC | ACGTTGGATGTCACACCATTTGACTTTTGC | GACTTTTGCAGACAGGCACA |
| **rs5745696** | ACGTTGGATGTCTGAATGAAGAGCTATGAG | ACGTTGGATGCATACAACACAAATCTCCCC | CAAATCTCCCCTCTCCTATGG |
| **rs5745627** | ACGTTGGATGTGAGGACCAATTGTAGAAGG | ACGTTGGATGATCAGTGCTAGAAGCATATC | GCTAGAAGCATATCATATCATG |
| **rs1207453** | ACGTTGGATGTAACACCCACAAAGAGGTGC | ACGTTGGATGGGCCTAATTGCAACCATTTG | AAATAAGGATTGTTAACTCTACA |
| **rs5745687** | ACGTTGGATGTGACACTGATGTTCCTTTGG | ACGTTGGATGAATGGTATTGACAGTGCCCC | CTTCTCCTTGACCTTGGATGCATT |
| **rs17155414** | ACGTTGGATGCCAATGAATTAAAACACAAG | ACGTTGGATGTATCCATAACTTTAAAATGTG | CCATAACTTTAAAATGTGTATAACT |
| **rs2286194** | ACGTTGGATGTTGTTATAGCTATTCTGAG | ACGTTGGATGCTAGAATTCTAGAATGTATGC | GCATGTTTATAAATAGAGTTCTAAC |
|  |  |  |  |

tSNP= tag single nucleotide polymorphism

PCR=polymerase chain reaction
