# Supplementary material for: Association of the Hepatocyte Growth Factor Gene with Keratoconus in an Australian Population
Source: PLoS One. 2014 Jan 8;9(1):e84067. doi: 10.1371/journal.pone.0084067 (PMC3885514; doi:10.1371/journal.pone.0084067)
Supplement: Table S2 — Raw data of genotyping frequency and tag single nucleotide polymorphisms call rate of the HGF gene. (DOCX) [file pone.0084067.s003.docx]

**Supplementary Table S2. Raw data of genotyping frequency and tag single nucleotide polymorphisms call rate of the *HGF* gene**

| **Genotyping rate** | **tSNP** | **TEST** | **Allele 1** | **Allele 2** | **Genotyping frequency** |
| --- | --- | --- | --- | --- | --- |
|  |  | ALL | G | A | 34/267/518 |
| **98.70%** | rs1019012 | Cases | G | A | 9/50/94 |
|  |  | Controls | G | A | 25/217/424 |
|  |  |  |  |  |  |
|  |  | ALL | G | A | 28/276/518 |
| **99%** | rs12707453 | Cases | G | A | 6/55/93 |
|  |  | Controls | G | A | 22/221/425 |
|  |  |  |  |  |  |
|  |  | ALL | A | C | 37/278/483 |
| **96.10%** | rs17155414 | Cases | A | C | 8/54/91 |
|  |  | Controls | A | C | 29/224/392 |
|  |  |  |  |  |  |
|  |  | ALL | A | T | 35/242/524 |
| **96.50%** | rs2286194 | Cases | A | T | 2/36/115 |
|  |  | Controls | A | T | 33/206/409 |
|  |  |  |  |  |  |
|  |  | ALL | A | G | 49/295/480 |
|  | rs5745616 | Cases | A | G | 8/63/83 |
| **99.30%** |  | Controls | A | G | 41/232/397 |
|  |  |  |  |  |  |
|  |  | ALL | C | G | 0/49/771 |
| **98.80%** | rs5745627 | Cases | C | G | 0/8/145 |
|  |  | Controls | C | G | 0/41/626 |
|  |  |  |  |  |  |
|  |  | ALL | A | G | 6/101/702 |
| **97.50%** | rs5745687 | Cases | A | G | 2/18/130 |
|  |  | Controls | A | G | 4/83/572 |
|  |  |  |  |  |  |
|  |  | ALL | C | G | 2/42/775 |
| **98.70%** | rs5745692 | Cases | C | G | 1/7/146 |
|  |  | Controls | C | G | 1/35/629 |
|  |  |  |  |  |  |
|  |  | ALL | A | G | 3/103/717 |
| **99.20%** | rs5745696 | Cases | A | G | 0/18/136 |
|  |  | Controls | A | G | 3/85/581 |
|  |  |  |  |  |  |
|  |  | ALL | A | G | 94/314/398 |
|  | rs5745752 | Cases | A | G | 32/52/58 |
| **97.10%** |  | Controls | A | G | 62/262/340 |
